# Production and distribution of chromosome aberrations in human lymphocytes by particle beams with different LET

**DOI:** 10.1007/s00411-018-0771-4

**Published:** 2019-01-17

**Authors:** Agata Kowalska, Elena Nasonova, Konrad Czerski, Polina Kutsalo, Wiktoria Pereira, Evgeny Krasavin

**Affiliations:** 1 Department of Physics and Chemistry, Maritime University of Szczecin, Wały Chrobrego 1-2, 70-500 Szczecin, Poland; 2 Institute of Physics, University of Szczecin, ul. Wielkopolska 15, 70-451 Szczecin, Poland; 3 Joint Institute for Nuclear Research, Joliot-Curie 6, 141980 Dubna, Russia

**Keywords:** Chromosome aberrations, Local energy deposition, Linear-quadratic model, Poisson distribution, Neyman A distribution

## Abstract

We investigated induction of chromosome aberrations (CA) in human lymphocytes when exposed to 150 MeV and spread out Bragg peak (SOBP) proton beams, and 199 MeV/u carbon beam which are currently widely used for cancer treatment and simultaneously are important components of cosmic radiation. For a comparison, the boron ions of much lower energy 22 MeV/u and a ^60^Co γ rays were used. Dose–effect curves as well as the distributions of CA were studied using Poisson and Neyman type A statistics. Systematics of experimentally determined parameters, their dependence on applied doses and irradiation quality are presented.

## Introduction

The study of biological efficiency of accelerated particle beams is of great interest in medicine due to the increasing use of hadron therapy for cancer treatment (Schulz-Ertner et al. 2007; Nikoghosyan et al. 2004). Furthermore, protons, carbon and other high energetic light ions are known to dominate in the space radiation flux (unlike terrestrial background radiation, dominated by γ radiation) and mainly determine the radiation hazard and health risks to astronauts during long space missions (Cucinotta and Durante 2006).

Chromosome aberrations (CA) are considered to be sensitive and reliable indicators of radiation action in humans and their assessment is a valuable method in biodosimetry. Induction of CA in human peripheral blood lymphocytes (PBL) was chosen as an endpoint of radiation exposure due to several reasons: (1) blood probes are the most simple and available samples of human tissues; (2) the fact that quiescent PBLs represent a population naturally synchronized in G_0_ facilitates the data interpretation; (3) PBL are actually used as a model of bone marrow cells (BMC) which are known to be the most sensitive cells of human organisms and are used as the limitation factor in health risk estimations.

Experimental studies devoted to CA as a response to ionizing radiation are often performed by means of dose–effect curves, which usually have a linear-quadratic shape (Lea 1955) and can be easily used for estimation of the clinically important relative biological effectiveness (RBE). The quadratic term, like to the nonlinear term in the survival curves, can result from two different effects. The first one is of a physical origin and arises from overlapping ion tracks at high ion fluencies, which locally lead to higher doses and a stronger biological response (Scholz 2006; Loucas et al. 2013). Generally, it is assumed that the spatially localized dose distribution within the ion tracks induced by heavy charged particles follows the distribution of fast electrons of the ionization process and, therefore, can be in principle calculated analytically. Otherwise, the quadratic term in the dose–effect curve can also result from the repair mechanisms of the DNA damage leading to CA, which also depends on the local ionization density and thus on the radiation quality (Scholz and Kraft 1996; Loucas et al. 2013).

Another effect that can be observed in radiobiological experiments is a considerable difference in frequency distributions of CA obtained for low and high LET radiations (Gudowska-Nowak et al. 2007). This is caused once again by differences in microscopic energy deposition of both radiation qualities. Uniform dose deposition of low-LET radiations (i.e., X-rays or γ quanta) results in a simple random damage distribution, well described by Poisson statistics. In comparison, microscopically inhomogeneous pattern of energy deposition characteristic for high-LET particles leads to clusters of damages randomly distributed along paths of ionizing particles (i.e., ion tracks). Since ions are also randomly distributed within the cell nucleus, the aberration frequency is well described by the Neyman type A statistics, which folds these two stochastic processes (Gudowska-Nowak et al. 2005, 2007; Deperas-Standyło et al. 2012). Such variable energy deposition results in higher frequency of cells carrying multiple aberrations, but also higher frequency of non-hit cells.

Generally, both kinds of studies can help us to separate physical and biological contributions to observed effects. However, there are as yet no ab initio calculations which could do it and, therefore, some phenomenological models have been developed concerning the highly inhomogeneous distribution of the dose deposited by heavy particle irradiation. One of the most used is the Local Effect Model (LEM) predicting the biological effect of ions from the response of cells and tissues to photon radiation (Scholz and Kraft 1996). Nevertheless, experimental data have shown some overestimation of the dose calculated for light ions, especially for protons (Scholz et al. 1997). The last version of the model, LEM IV (Friedrich et al. 2012) includes a microscopic double-strand distribution of DNA, including a giant loop structure. It allows to calculate the RBE values much more precisely but at the cost of introducing an additional free parameter. Some problems with understanding of the proton interaction with biological samples in terms of the low RBE values have been previously discussed (Kowalska et al. 2015) and it was suggested that the local dose is possibly distributed in a larger region than only within the standard ion track. Another model, which assumes the amorphous track structure of heavy ions, has been developed by Katz (Katz et al. 1971). The model distinguishes between two action modalities “ion-kill” and “γ-kill”. There is an additional contribution at high fluencies where δ-electrons of several ions can overlap in space and determine the additional effect (γ-kill). The model of Katz is well suited to reproduce the cell-survival curves and the Z-dependence of the RBE-LET function including the overkill effects; therefore, both physical and biological processes might be simultaneously described.

The purpose of our work is to propose a new analytical model that could use experimentally determined dose–effect curves and CA statistical distributions to conclude the effective biological interaction radius within the ion tracks and compare the obtained values with physical predictions. For our study, we collected experimental data using several particle species of different LET values which are of great importance in the space research and therapy: the 150 MeV proton beam and the SOBP (spread out Bragg Peak) protons as well as the 199 MeV/u ^12^C and 22 MeV/u ^11^B beams.

Here, we will focus on a more detailed analysis of experimental results and on determining values of representative parameters of the dose–effect curves and of aberration frequency distributions. We would like to demonstrate the differences in experimentally determined parameters for different radiation qualities and also arrive at a data set suitable for a modelling study that we plan to perform.

## Materials and methods

### Blood samples and irradiation

The blood used for the study was obtained by venipuncture into heparinized vacuum containers. The samples were collected from informed, healthy volunteers, in accordance with local ethical regulations. The whole blood was irradiated in 0.5 ml Eppendorf tubes; whereas isolated lymphocytes used for the boron irradiation were placed in special Plexiglas vessels. All exposures were done at room temperature and controls were sham-irradiated. The scoring and recording criteria followed those given in IAEA Manuals (2001, 2011). Irradiation experiments were performed for each quality separately.

### Proton beam

Proton exposure was performed at the clinical proton beam facility of the Medico-technical complex of Dzhelepov Laboratory of Nuclear Problems, JINR, Dubna, Russia (for more details see Pachnerova Brabcova et al., 2014; Racjan et al., 2015). Blood samples were irradiated with unmodified 150 MeV proton beams (LET 0.57 keV/µm) and with slowed down protons at the central region of the 10 mm wide SOBP plateau at an experimentally determined average LET 1.4 keV/µm (Kubancak and Molokanov 2013). Dose rate in the target volume amounted to 0.7 Gy/min for high energy protons and 1.3 Gy/min in the SOBP. As a reference, the ^60^Co γ radiation source of the radiation therapy unit ROKUS-M was used. Dose rate at irradiation point was 0.82 Gy/min. Doses ranged between 1 and 5 Gy for protons and 0.5–3 for ^60^Co γ-rays.

### Carbon beam

Irradiation with 199 MeV/u ^12^C ions (LET 16 keV/µm) was done at the ITEP-TWAC accelerator (Russia, Moscow). Special features of the beam extraction from the synchrotron enabled us to use short ion pulses with duration of 500 ns. Irradiation was carried out in the plateau region of the Bragg curve, where the LET of the particles did not change significantly (Markov et al. 2014). Doses were ranging between 0.8 and 6.37 Gy.

### Boron beam

The PBL were irradiated with doses of 0.05–2 Gy applying the monoenergetic ^11^B beam of energy 22.1 MeV/u (average LET 76 keV/µm) generated in the MC-400 cyclotron at Flerov Laboratory of Nuclear Reactions, JINR, Dubna, Russia. The isolated lymphocytes were highly concentrated in nutrient medium and irradiated as 1.5 mm layer in specially designed Plexiglas chambers sealed by 8 µm polycarbonate foils so that all ions passed the sample and stopped behind it. The chambers were exposed using automatic irradiation facility Genom-M (Bezbakh et al. 2013).

### Cell cultivation and metaphase analysis

Immediately after ^11^B irradiation, isolated lymphocytes were seeded with a density of 0.5 × 10^6^/ml in RPMI medium supplemented by 20% 6. fetal calf serum, 2 mM l-glutamine, 100 U/ml penicillin, 100 µg/ml streptomycin and 1.5% phytohaemagglutinin (PHA).

After exposure to proton, carbon and ^60^Co γ rays, the blood samples were diluted in 4.5 ml of the same medium. All samples were incubated at 37 °C and 5% CO_2_. Cells were fixed at 48 h after PHA stimulation proceeded by 3 h colcemid treatment (200 ng/ml) for metaphase accumulation and stained in 3% Giemsa. Typically, 100–300 metaphases were analyzed for every data point. Chromosomal aberrations were classified according to (Savage 1975). All aberrations of the chromosome and chromatid types visible without karyotyping were recorded. The chromosome-type aberrations comprise paired fragments, dicentrics, centric and acentric rings (the latter also includes double minutes) and translocations visible without karyotyping. The minor fraction of chromatid-type aberrations includes the chromatid-type breaks and chromatid-type exchanges. The gaps were not scored as aberrations.

### Statistical analysis, distribution of aberrations

Statistical distribution of the number of observed CA can be described by two different stochastic distributions: Poisson and Neyman A. The Neyman A distribution is a folding of two independent Poisson distributions. One of them ${P_{{\lambda _{\text{N}}}}}$(*n*) describes the probability that the cell nucleus will be hit by *n* ions. The other Poisson probability ${P_{n\mu }}$(*k*) assess the number of aberrations *k* produced by each hit (Gudowska-Nowak et al. 2007):

1 $$\begin{aligned}{P_{\text{N}}}\left( m \right)&=\mathop \sum \limits_{{n=0}}^{\infty } {P_{n\mu }}\left( k \right){P_{{\lambda _{\text{N}}}}}\left( n \right)\\&=\mathop \sum \limits_{{n=0}}^{\infty } \frac{{{{(n\mu )}^k}{e^{ - n\mu }}}}{{k!}} \cdot \frac{{\lambda _{{\text{N}}}^{n}{e^{ - {\lambda _N}}}}}{{n!}} =\frac{{{\mu ^k}}}{{k!}}\mathop \sum \limits_{{n=0}}^{\infty } \frac{{{n^k}}}{{n!}}{({e^{ - \mu }}{\lambda _{\text{N}}})^n}\end{aligned}$$

Here *λ*_N_ and *µ* reflect the mean number of particle traversals per cell and mean number of CA induced by a single hit, respectively. Parameter *λ*_N_ can be calculated from the particle fluence and the cross section of human lymphocytes of ~ 25 µm^*2*^ (Anderson et al. 2000), which leads to the fluence of 4 × 10^6^ particles/cm^2^ corresponding to one hit per cell. The variance of the Neyman A distribution is larger than its mean value and can be expressed as: $\sigma _{{\text{N}}}^{2}={\lambda _{\text{N}}}~\mu \left( {1+\mu } \right)$ (Gudowska-Nowak et al. 2007).

In the case of low-LET radiation, when *µ* is very low, the variance corresponds to that of a Poisson distribution as for γ radiation. Nevertheless, the energy distribution imparted by many low-LET particles due to their ion track structure still differs from that of γ quanta, which is almost homogenously distributed. For the simple Poisson statistics, the aberration frequency can be calculated as follows:

2 $${P_{\text{p}}}\left( m \right)=\frac{{\lambda _{{\text{P}}}^{m}~{e^{ - {\lambda _{\text{P}}}}}}}{{m!}}$$

Here *m* stands for the number of aberrations per individual cell and *λ*_P_ is the average number of CA observed in the whole cell population exposed to a given dose of a given radiation. Parameters *λ*_P_, *µ* and *λ*_N_ are linked by a simple relation: ${\lambda _{\text{P}}}={\lambda _{\text{N}}} \cdot \mu$. Mean numbers of hits per nucleus for a given dose of a given ion irradiation are presented in Table 2.

Cytogenetic data are distributed according to Poisson statistics when relative variance ${\sigma ^2}/\left\langle X \right\rangle$ is equal to one (Edwards et al. 1979) where $\left\langle X \right\rangle$ denotes the experimentally determined mean number of CA per cell. It results from the fact that for the Poisson distribution $\left\langle X \right\rangle$ is equal to *λ*_P_ and $\left\langle {{X^2}} \right\rangle =~\lambda _{{\text{P}}}^{2}+{\lambda _{\text{P}}}$, thus the variance $\sigma _{{\text{P}}}^{2}$ is equal to the mean value:

3 $$\sigma _{{\text{P}}}^{2}=\left\langle {{X^2}} \right\rangle - {\left\langle X \right\rangle ^2}=\lambda _{{\text{P}}}^{2}+{\lambda _{\text{P}}} - \lambda _{{\text{P}}}^{2}={\lambda _{\text{P}}}$$

To judge whether a deviation from the Poisson statistics is significant, the so-called *U* test has been used (Edwards et al. 1979). The *U* test gives a normalized comparison of the relative variance with the expected Poisson value at the 95% confidence level. Distributions for which the *U* test values are smaller than − 1.96 or larger than + 1.96 are under- or over-dispersed compared to the Poison distribution, respectively.

The formula to assess values of the *U* test reads as follows:

4 $$U=\frac{{d - (N - 1)}}{{\sqrt {\sigma _{d}^{2}} }},$$

where *N* is the number of analyzed metaphases, *d* represents the coefficient of dispersion which provides an indicator of how well the variance of a given sample corresponds to the Poisson distribution. The coefficient of dispersion and its corresponding variance can be calculated according to the following equations:

5 $$d=\frac{{(N - 1)\sigma _{{\text{P}}}^{2}}}{{{\lambda _{\text{P}}}}},\quad \sigma _{d}^{2}=2(N - 1)(1 - 1/N{\lambda _{\text{p}}})$$

## Results

**Fig. 1:**
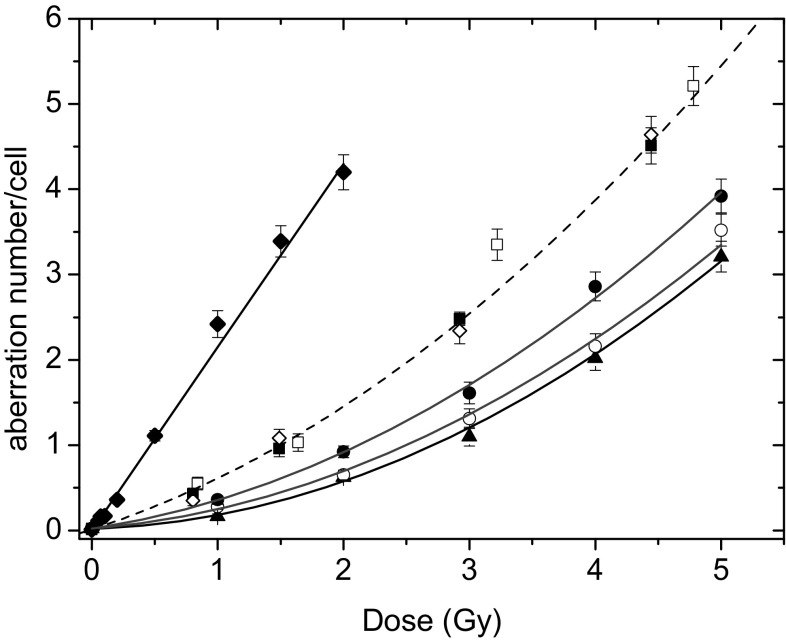
Dose–effect curves plotted for chromosome aberrations produced by all radiation species listed in Table 2: (filled triangle) ^60^Co γ rays, LET 0.2 keV/µm; (open circle) high energy protons 150 MeV, LET 0.57 keV/µm; (filled circle) SOBP protons, LET 1.4 keV/µm; (open square—donor 1, filled square—donor 2, open diamond—donor 3) ^12^ C ions 199 MeV/u, LET 16.3 keV/µm; (filled diamond) ^11^B ions 22.1 MeV/u, LET 76 keV/µm. Error bars are calculated according to Poisson statistics: $\Delta y=\sqrt Y /N$, where *Y* is the total aberration number and *N* is the number of cells scored for each point

The percentage of aberrant cells, total CA yield and CA spectra produced by all radiation species used are listed in Table 2, and the dose-dependence of CA frequencies is depicted in Fig. 1. The total CA numbers have been fitted by a linear-quadratic function with exception of those induced by boron ions, which were fitted by a linear relation. Parameters of the least squares fits are presented in Table 1.

**Table 1:** Parameters of the dose–effect curve fitting

Beam, LET	*α* (Gy ^−1^)	*β* (Gy^−2^)	*β*/*α* (Gy^−1^)
^11^B ions, 76 keV/µm	2.15 ± 0.06	–	–
^12^C ions, 16 keV/µm	0.48 ± 0.06	0.12 ± 0.01	0.25 ± 0.05
SOBP protons, 1.4 keV/µm	0.22 ± 0.04	0.11 ± 0.01	0.5 ± 0.14
High en. protons, 0.57 keV/µm	0.12 ± 0.03	0.11 ± 0.01	0.92 ± 0.31
^60^Co γ rays, 0.2 keV/µm	0.05 ± 0.03	0.12 ± 0.01	2.40 ± 1.64

**Table 2:** Frequency of CA induced in PBL by ^60^Co γ rays, high energy protons, SOBP protons, ^12^C ions and ^11^B ions (*ctb* chromatid breaks, *csb* paired fragments, *dic* dicentrics, *Race* acentric rings, *Rc* centric rings, *trans* translocations, *cte* chromatid exchanges)

Irradiation	Dose, Gy	Mean no. of hits/cell (*λ*_N_)	No. of cells scored	Aberrant cells (%)	Aberrations per 100 cells						Sum of aberrations /100 cells	Aberrations /aberrant cells
					ctb	csb	R ac	Dic	Rings	trans/cte		
^60^Co $\gamma$ rays	0	–	200	2	1	1	0	0	0	0	2	1.0
	1	–	200	15.5	1	3	2	9.5	1	0	16.5	1.1
	2	–	300	48.7	2.3	10	5.7	38.7	5.3	0.3/0	62.3	1.3
	3	–	100	65	2	23	11	57	4	3/2	102	1.6
	4	–	100	83	4	38	31	113	11	5/0	202	2.4
	5	–	100	97	3	54	42	195	20	6/1	321	3.3
High energy protons	0	0	200	2	1	1	0	0	0	0	2	1.0
	1	274	300	24.7	1.7	8.3	2.7	12	2.3	0.3/0	27.7	1.1
	2	548	200	45	0.5	13	6.5	39	5.5	0.5/0	65	1.4
	3	821	100	74	2	16	23	73	15	2/0	131	1.8
	4	1096	100	89	3	31	30	127	21	4/0	216	2.4
	5	1369	100	98	5	65	45	207	25	5/0	352	3.6
SOBP protons	0	0	200	2	1	1	0	0	0	0	2	1.0
	1	126	200	28	1	8	3.5	20	3.5	0	36	1.3
	2	252	200	67.5	3	12	5.5	61	7.5	3/0	92	1.4
	3	378	100	77	0	19	18	106	12	4/0	161	2.1
	4	504	100	94	4	54	27	165	31	4/1	286	3.0
	5	630	100	97	5	83	41	223	33	6/1	392	4.0
^12^C ions donor 1, filled square	0	0	100	2	2	0	0	0	0	0	2	1.0
	0.84	8	100	39	7	13	6	23	5	1/0	55	1.4
	1.64	15.8	200	64.5	4.5	29	10	52	6	1/0.5	103	1.6
	3.22	30.8	100	98	5	114	30	143	35	8/0	335	3.4
	4.78	45.8	100	99	8	181	31	258	36	7/0	521	5.3
	6.37	61	100	100	14	246	75	393	63	15/0	806	8.1
^12^C ions donor 2, open square	0	0	100	1	1	0	0	0	0	0	1	1.0
	0.8	7.6	100	33	0	13	1	24	4	1/0	43	1.3
	1.49	14.2	100	57	3	31	6	48	6	2/0	96	1.7
	2.92	27.8	200	88.5	2.5	83	17	122.5	19	3.5/0/5	248	2.8
	4.44	42.3	100	96	9	134	29	231	42	4/2	451	4.7
	5.89	56	100	100	7	230	65	341	45	9/1	698	7.0
^12^C ions donor 3, open diamond	0	0	100	1	1	0	0	0	0	0	1	1.0
	0.8	7.62	100	27	1	5	4	18	5	2/0	35	1.3
	1.49	14.2	100	64	2	25	10	62	7	2/0	108	1.7
	2.92	27.81	100	87	8	69	14	128	12	3/0	234	2.7
	4.44	42.29	100	99	11	126	37	253	27	10/0	464	4.7
	5.89	56.1	100	100	3	198	51	349	56	13/2	672	6.7
^11^B ions	0	0	100	1	1	0	0	0	0	0	1	1.0
	0.05	0.11	200	8	1	2	0.5	7.5	0.5	0	11.5	1.4
	0.07	0.15	100	14	2	4	2	8	0	0	16	1.1
	0.1	0.22	200	10.5	1.5	5.5	3.5	7	0	0	17.5	1.7
	0.2	0.43	200	20.5	2.5	9	8.5	13.5	2.5	0.5/0	36	1.8
	0.5	1.07	200	53.5	6	25	19.5	60	5	1/0	116.5	2.2
	1	2.2	100	71	9	66	30	115	20	1/1	242	3.4
	1.5	3.2	100	82	10	102	62	151	13	1/0	339	4.1
	2	4.3	100	83	8	141	84	149	34	4/0	420	4.1

Additionally, mean number of hits per cell (*λ*) for each ion irradiation is appended

The spectra of CA are very similar for all low-LET radiations (photons and both proton beams) where exchange-type aberrations comprise 75–85% of total CA yield while these values decrease down to 64–70% for carbon and boron ions.

**Fig. 2:**
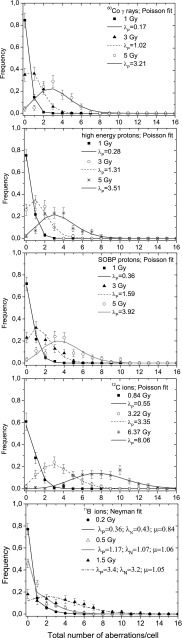
Distributions of CA frequencies per cell

**Fig. 3:**
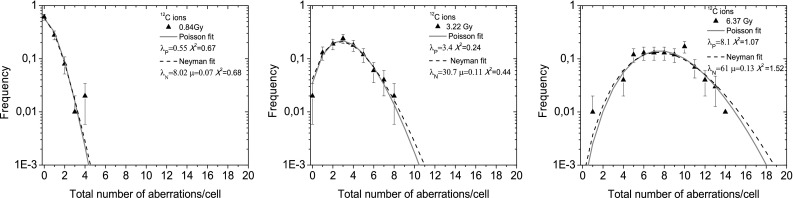
CA frequencies induced by ^12^C ions fitted by the Poisson and Neyman A distributions. The goodness of fit was verified by the Chi square test

**Fig. 4:**
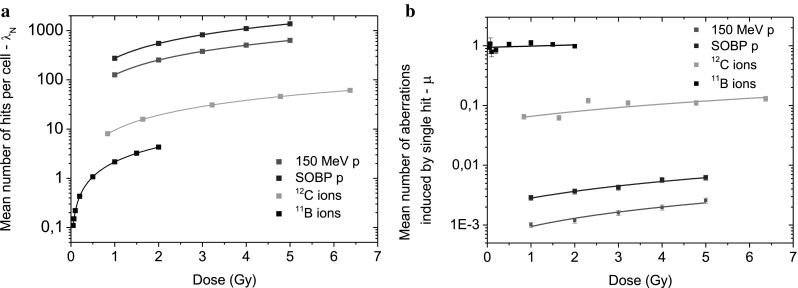
**a** Dose-dependence of the *λ*_N_ parameter (describing mean number of hits per cell) obtained for high energy protons, ^12^C ions and ^11^B ions. **b** Dependence of the parameter *µ* (describing the mean number of aberrations induced by single ion hit) on the radiation dose determined for high energy protons, ^12^C ions and ^11^B ions

Distributions of CA frequencies have been analyzed for radiation of different quality. Data obtained for ^60^Co γ - rays, high energy protons, SOBP protons and carbon ions could be fitted either by the Poisson or by the Neyman A distribution whereas the data obtained for ^11^B irradiation could be analyzed only by means of the Neyman A distribution. Accordingly, experimentally determined CA distributions and corresponding fits for chosen doses of each irradiation type are shown in Fig. 2. For comparison, CA spectra obtained for carbon irradiation were fitted by means of both statistical models (Fig. 3). The dose-dependence of the parameters *λ*_N_ and *µ*, describing the mean number of hits per cell and of aberrations induced by single ion hit, respectively, obtained for high energy and SOBP protons, ^12^C ions and ^11^B ions are depicted in Fig. 4a, b.

We have also estimated the average number of particle hits *λ*_N_ per spherical G0 lymphocyte nucleus of diameter ~ 5 µm. At 1 Gy it amounts to 274 in case of 150 MeV protons and 126 in case of SOBP protons, while for carbon and boron ions, it amounts only to 10.2 and 2.15, respectively. In the last case, it means that according to the Poisson statistics, 12% of cells received no hit, 25%—1 hit, 27%—2 hit, 36%—3 and more hits/nucleus.

When comparing both distributions for the same mean number of aberrations per cell, Neyman A gives a higher number of non-hit cells and a higher number of cells with multiple aberrations. This statistical prediction can be demonstrated by a comparison between the Poisson distribution fitted for 3 Gy of high energy protons (*λ*_P_ = 1.32) and the Neyman A fitted for 0.5 Gy boron irradiation (*λ*_P_ = 1.13). In the case of 150 MeV protons, the frequency of “zero-class” cells is 0.33 ± 0.05 and for boron ions it amounts to 0.46 ± 0.03.

Differences between both distributions are also detectable in the number of cells carrying multiple aberrations. For example, for the 2 Gy boron irradiation with the mean number of aberrations per cell *λ*_P_ = 4.2, the frequency of cells carrying eight aberrations amounts to 0.08 ± 0.03. The dose of 5 Gy of fast protons (*λ*_P_ = 3.5) results in the much lower frequency of cells carrying eight aberrations of 0.01 ± 0.01. It means that among one hundred of scored cells (exposed to the dose leading to the mean number of ~ 4 aberrations per cell), eight cells carried eight aberrations in case of boron ions and only one cell in case of 150 MeV protons.

Evaluation of the degree of spread of experimental data has been also performed by assessing the variance to mean ratio $({\sigma ^2}/\left\langle X \right\rangle )~$ and the *U* test (see Table 3). The relative dispersion has been found significantly higher than one in the case of ^11^B ions. In the case of ^60^Co γ-rays and high energy proton beam, the relative dispersion is lower than one; however, such under-dispersion is not significant according to the applied *U* test. Only for 0.84 Gy ^12^C ions, the relative variance amounting to 1.29 is at the edge of significance (see Table 3). The *U* test delivers, however, both negative and positive values for different doses.

**Table 3:** Expected values of the aberration number per cell *λ*_P_, ion hits per cell *λ*_N_ and the aberration number induced by each hit *μ* after exposure to protons, carbon and boron ions

	Dose (Gy)	Mean number of aberrations per cell (*λ*_P_)	Mean number of hits per cell (*λ*_N_)	Mean number of aberrations/hit (µ)	Dispersion (*σ*^2^)	Relative Dispersion (*σ*^2^/<*X*>)	*U* test
^60^Co γ-rays	1	0.17 ± 0.03	–	–	0.16	0.96	− 0.44
	3	1.02 ± 0.10	–	–	0.90	0.88	− 0.83
	5	3.2 ± 0.2	–	–	2.2	0.68	− 2.3
High energy protons	1	0.28 ± 0.03	274 ± 14	1.0 × 10^−3^ ± 0.1 × 10^−3^	0.26	0.94	− 0.73
	3	1.31 ± 0.11	821 ± 41	1.6 × 10^−3^ ± 0.2 × 10^−3^	1.17	0.90	− 0.73
	5	3.5 ± 0.2	1369 ± 68	2.6 × 10^−3^ ± 0.3 × 10^−3^	2.8	0.79	− 1.5
SOBP protons	1	0.36 ± 0.04	126.6 ± 6	2.9 × 10^−3^ ± 0.3 × 10^−3^	0.42	1.76	1.69
	3	1.59 ± 0.13	378 ± 17	4.3 × 10^−3^ ± 0.4 × 10^−3^	1.74	1.09	0.63
	5	3.9 ± 0.2	630 ± 28	6.2 × 10^−3^ ± 0.6 × 10^−3^	3.0	0.77	− 1.62
^12^C ions donor 1	0.84	0.55 ± 0.07	8.0 ± 0.4	0.07 ± 0.01	0.71	1.29	2.03
	3.22	3.35 ± 0.18	30.7 ± 1.5	0.11 ± 0.01	2.9	0.86	− 0.98
	6.37	8.1 ± 0.3	61 ± 3	0.13 ± 0.01	6.0	0.75	− 1.77
^11^B ions	0.2	0.36 ± 0.04	0.43 ± 0.02	0.84 ± 0.10	0.61	1.70	7.0
	0.5	1.17 ± 0.08	1.07 ± 0.05	1.06 ± 0.09	2.0	1.75	9.1
	1.5	3.4 ± 0.2	3.2 ± 0.2	1.05 ± 0.08	7.4	2.2	8.4

Values of the relative dispersion and the *U* test are also presented. Analysis was done for several chosen doses

According to the Neyman A distribution, we would expect a constant value of the parameter *µ*—independent of the dose. It is well fulfilled for boron ions, having relatively high ionization density. Values of the *µ* parameter obtained for boron ions are scattered around its mean 0.94 ± 0.06, i.e., every particle hit induced a chromosome aberration. An increase of the *µ* value with the dose is, however, observed for particles with lower LET: carbon ions, SOBP and high energy protons for which is the most pronounced (see Table 4).

**Table 4:** Fitting parameters of the dose-dependence of the *λ*_N_ and *µ*

Particle	*λ*_N_ (D)		*µ* (D)	
	Int.	Slope	Int.	Slope
High energy protons	− 2.1 ± 9.5	275 ± 9	(5.9 ± 1.3) × 10^−4^	(3.5 ± 0.5) × 10^−4^
SOBP protons	(− 8.75 ± 23.5) × 10^−4^	(126 ± 9) × 10^−4^	(19.7 ± 3.6) × 10^−4^	(8.5 ± 1.4) × 10^−4^
^12^C ions	− 0.04 ± 0.56	9.58 ± 0.35	0.053 ± 0.006	0.013 ± 0.002
^11^B ions	(2.0 ± 1.4) × 10^−3^	2.145 ± 0.002	0.94 ± 0.06	0.05 ± 0.05

The second parameter *λ*_N_, corresponding to the mean number of hits per single cell, is determined from the projectile fluence *F* applying the simple relation $${\lambda _{\text{N}}}=\sigma ~ \times F,$$ where $$\sigma$$ corresponds to the cross section area of our target (25 µm^2^), and the fluence is related to the dose: $$D=1.602 \times {10^{ - 9}} \times LET \times F \times {\raise0.7ex\hbox{$1$} \!\mathord{\left/ {\vphantom {1 \rho }}\right.\kern-0pt}\!\lower0.7ex\hbox{$\rho $}}$$ where *ρ* is the target mass density. As expected, the dose-dependence of *λ*_N_ is linear (see Fig. 4a; Table 4).

## Discussion and conclusions

Chromosome aberrations (CA) are considered to be the most sensitive and reliable bioindicator of radiation action. The detailed analysis of chromosome aberrations as an endpoint of radiation exposure is thus essential for biodosimetric considerations and for risk of carcinogenesis assessment (Bonassi et al. 2004). The importance of such studies is forced not only by the wide use of hadron therapy for cancer treatment, but also by the necessity of the astronaut protection during space missions (Schulz-Ertner et al. 2007; Cuccinotta and Durante 2006).

In this study, we investigated CA induction in human lymphocytes by different heavy charged projectiles (protons, ^12^C and ^11^B ions) and compared it with the results obtained for ^60^Co γ rays. The data with the exception of those obtained for boron ions were collected for different patients in relation to our previously published study (Kowalska et al. 2015) to reduce experimental uncertainties. Both, dose–effect curves and statistical distributions of CA were used to analyze the dependence of experimentally determined parameters on the radiation quality which will be compared with predictions of different radiobiological models in the second part of the work.

The experimental dose–effect curves have a linear-quadratic shape for all radiations applied with exclusion of ^11^B ions for which a linear dependence is observed (see Table 1). This simple linear behavior is usually found for high-LET radiation qualities (Lee et al. 2005) and should not be interpreted as a vanishing of the quadratic parameter *β*. It is rather the result of a strong increase of the linear parameter *α* for high-LET values corresponding to a higher ionization density. Since our LET values are still below the maximum of the biological effectiveness expected for LET of about 100 keV/µm, the *α* parameter increases almost linearly (Ando and Goodhead 2016). Therefore, the linear part of the dose–effect curve for ^11^B ions should dominate the quadratic term and reduces the curvature of the response function.

On the other hand, the quadratic parameter reflects, as mentioned before, radiobiological repair mechanisms and physical effects of overlapping ionization areas of individual projectiles at higher fluencies. Thus, the study of LET dependence of the *β* parameter can contribute to understanding of both processes. In the present work, we have observed for the first time that the *β* values within the experimental uncertainties do not change significantly for different radiation qualities and LET values. Similar results, but obtained only for proton beams of different energies, has been recently reported (Wilkens and Oelfke 2004).

Differences in acting of ions of low- and high-LET ions can be also observed in the statistical distributions of CA. Neglecting the fact that the high-LET radiations produce more complex chromosome aberrations (Kowalska et al. 2017) and treating all aberration types similarly, the frequency of CA can be described by the Poisson as well as by Neyman type A distributions. The latter one includes effects of limited ionization area induced by heavy charged particles in the biological materials (so-called ion tracks). Whereas the Neyman A distribution parameter *λ*_N_ can be directly determined from the fluence of the applied radiation, the second one, *µ*, corresponds to the probability of CA production and is important for modelling of the interaction process. We have found that the differences between the Poisson and Neyman A distributions for low-LET radiation are very small. This result can be easily understood in terms of relation between the Poisson $\sigma _{{\text{P}}}^{2}$ and Neyman A $\sigma _{{\text{N}}}^{2}$ variances

6 $$\sigma _{N}^{2}=\sigma _{P}^{2}\left( {1+\mu } \right)$$

For a small value of the *µ* parameter (*µ* << 1), both distributions have similar variances. In our case, only ^11^B ions deliver *µ* values close to unity. Thus, the Poisson and Neyman A statistics strongly differ and give different shapes of the CA frequency distributions.

Another of our finding is related to the beam energy dependence of the determined *µ* parameter. Its value increases linearly with the energy of ions, and this increase is weaker for particles with higher LET values, vanishing finally for ^11^B ions (see Fig. 4 b; Table 4). Comprehensive model calculations including physical and radiobiological effects should certainly explain this finding.

To compare experimental CA frequencies with the Poisson statistics, we additionally applied the *U* test introduced by Edwards et al. (1979). It can be used to answer the question whether the frequency number of multiple aberrations is under- or overestimated compared to the Poisson distribution. Whereas a sign of underestimation can be found for γ rays and high energy protons (however, with a confidence level lower than 95%), only a significant overestimation can be observed for boron ions. The overestimation certainly comprises the main feature of the Neyman A distribution which increases the number of multiple aberrations. On the other hand, the underestimation can be explained by a contribution of repair effects (Kowalska et al. 2015), which is, however, very difficult to observe in the distribution of CA frequencies. It seems that the underestimation effect can be determined much more easily by the so-called Fano factor applying a Chi-square analysis as proposed in (Kowalska et al. 2015).

Summarizing, the present experimental study of the CA frequencies and resulting dose–effect curves obtained for different radiation qualities have demonstrated applicability of the Neyman A statistics even to low-LET irradiations. We have found some LET dependences of experimentally determined parameters of the dose effect curves and the Neyman A distributions which can be used for comparison with the radiobiological models.
